# Extracellular Vesicles, New Players in Sepsis and Acute Respiratory Distress Syndrome

**DOI:** 10.3389/fcimb.2022.853840

**Published:** 2022-04-07

**Authors:** Wenqiang Jing, Huijuan Wang, Liying Zhan, Wei Yan

**Affiliations:** ^1^ Department of Critical Care Medicine, Renmin Hospital of Wuhan University, Hubei Key Laboratory of Cell Homeostasis, College of Life Sciences, Wuhan University, Wuhan, China; ^2^ Department of Critical Care Medicine, Renmin Hospital of Wuhan University, Wuhan, China

**Keywords:** sepsis, ARDS, extracellular vesicles, diagnosis, engineering

## Abstract

Sepsis refers to a complex syndrome associated with physiological, pathological, and biochemical abnormalities resulted from infection. Sepsis is the major cause of acute respiratory distress syndrome (ARDS). Extracellular vesicles (EVs) are serving as new messengers to mediate cell-cell communication *in vivo*. Non-coding RNAs, proteins and metabolites encapsulated by EVs could result in either pro-inflammatory or anti-inflammatory effects in the recipient cells. Pathogens or host cells derived EVs play an important role in pathogens infection during the occurrence and development of sepsis and ARDS. Additionally, we summarize the potential application for EVs in diagnosis, prevention and treatment for sepsis and ARDS.

## Introduction

Sepsis is defined as life-threatening organ dysfunction caused by a dysregulated hosts response to infection (Singer et al., 2016; Cao et al., 2019). There are 48.9 million cases diagnosed every year, and mortality number is up to 11 million (Evans, 2018). Current management of sepsis is supportive, aiming at controlling the infection by timely using appropriate antimicrobial medicine, maintaining blood pressure by fluid resuscitation and vasopressors, and mechanical support of failing organs (Evans, 2018; Vandewalle et al., 2021). However, current therapeutic methods could not completely prevent the progression of sepsis. The pathogenesis of sepsis hasn’t been clearly clarified yet, and there is lack of specific methods and drug-treating targets in clinical practice. Immune regulation disorder underlies the pathophysiology of sepsis. Some patients mount a productive immune response to fight infection, whereas others deteriorate into a dysregulated state. Other pathophysiological changes involve dysregulated coagulation, aberrant mediator production, including hyperinflammatory response and blunted inflammatory response, cellular dysfunction, including lymphocyte apoptosis, neutrophil hyperactivity and endothelial cell failure and apoptosis in other cells and metabolic alterations, including insulin resistance, hyperglycemia and low-dose steroids. The pathogenesis of sepsis mainly covers four critical processes: endothelial dysfunction, coagulation abnormalities, alterations in cell functions and dysregulated cardiovascular responses (Evans, 2018).

The widespread organ dysfunction caused by sepsis involves lung, liver, kidney and brain (Faix, 2013), and sometimes influences multiple organs at the same time. In the cardiovascular system, hypotension occurs due to sepsis-induced pathologic arterial and venodilation. Additionally, the myocardium is damaged as well. Acute kidney injury is a leading cause for the death of septic patients. Severe sepsis and septic shock could even result in encephalopathy. Liver failure is an uncommon but significant complication of septic shock, with a severe impact on morbidity and mortality. Hematologic damage due to sepsis includes anemia, leukocytosis, neutropenia, thrombocytopenia, and disseminated intravascular coagulation (DIC). Many patients died of multiple organ dysfunction syndrome (MODS) or multiple organ failure (MOF). The survived patients couldn’t avoid suffered from serious complications including pulmonary fibrosis, neuromuscular weakness, and cognitive impairment persist even after treatment and recovery. Among them, lung is the most-easily injured organ during the occurrence of sepsis. The abnormal host response to infection leads to the destruction of the pulmonary alveolar-capillary barrier, resulting in acute lung injury characterized by hypoxemia, inflammation, and non-cardiogenic pulmonary edema (Englert et al., 2019). Once getting worse, lung injury would develop into ARDS with the mortality rate up to 40% (Englert et al., 2019). ARDS is the earliest and most common complication of sepsis. The pathophysiology mechanism of ARDS is mainly derived from the excessive transepithelial neutrophils migration, release of pro-inflammatory cytokines, fibro-proliferation, and activation of apoptosis, resultant loss of alveolar-capillary membrane integrity, active innate immune response, and unusual coagulation, which consequently induced the impairment of gas exchange function resulting in hypoxemia, reduced carbon dioxide excretion, and ultimately acute respiratory failure and the death of patients (Bao et al., 2015; Huppert et al., 2019; Chen et al., 2020). Further studies are needed focused on pathogenesis of sepsis and ARDS and identification of potential biomarkers as well as treatment strategies.

EVs with vesicle-like structures and double layer membranes, are widely existing in fluids such as blood, urine, saliva. According to their size, biogenesis, contents and functions, EVs were mainly divided into exosomes (40-160 nm), micro-vesicles (200-1000 nm), apoptotic bodies (500-2000 nm) (Akers et al., 2013; Shao et al., 2018; Doyle and Wang, 2019; Latifkar et al., 2019). EVs are carrying a variety of bioactive cargos, such as nucleic acids, proteins and lipids. Since discovered over one hundred years ago, EVs were initially considered as delivering garbage. However, more literatures were evidencing that EVs were functioning as diagnostic biomarkers in more diseases including tumor, infectious diseases, autoimmune diseases, neurodegenerative diseases, organ transplantation rejection, kidney and endocrine diseases. Moreover, they can also be used as therapeutic strategies in the repair and regeneration of injured tissues and organs (Shao et al., 2018; Kalluri and LeBleu, 2020). During multiple distinct ways of interaction between pathogens and host cells, as carriers for carrying signal molecules, EVs are playing fundamental role between occurrence and development of diseases. Therefore, we speculate that during the pathogenesis of sepsis and sepsis-induced ARDS, the transmission of infection-related molecules in the blood, and the signal transmission between injured organs might be related to EVs transfer. In this review, we will discuss the role of EVs in sepsis and sepsis-induced ARDS based on the existing studies, aiming to discover the diagnostic and therapeutic value of EVs in the diseases.

## The Biogenesis Of EV

### Biogenesis of Exosomes

**Figure 1:**
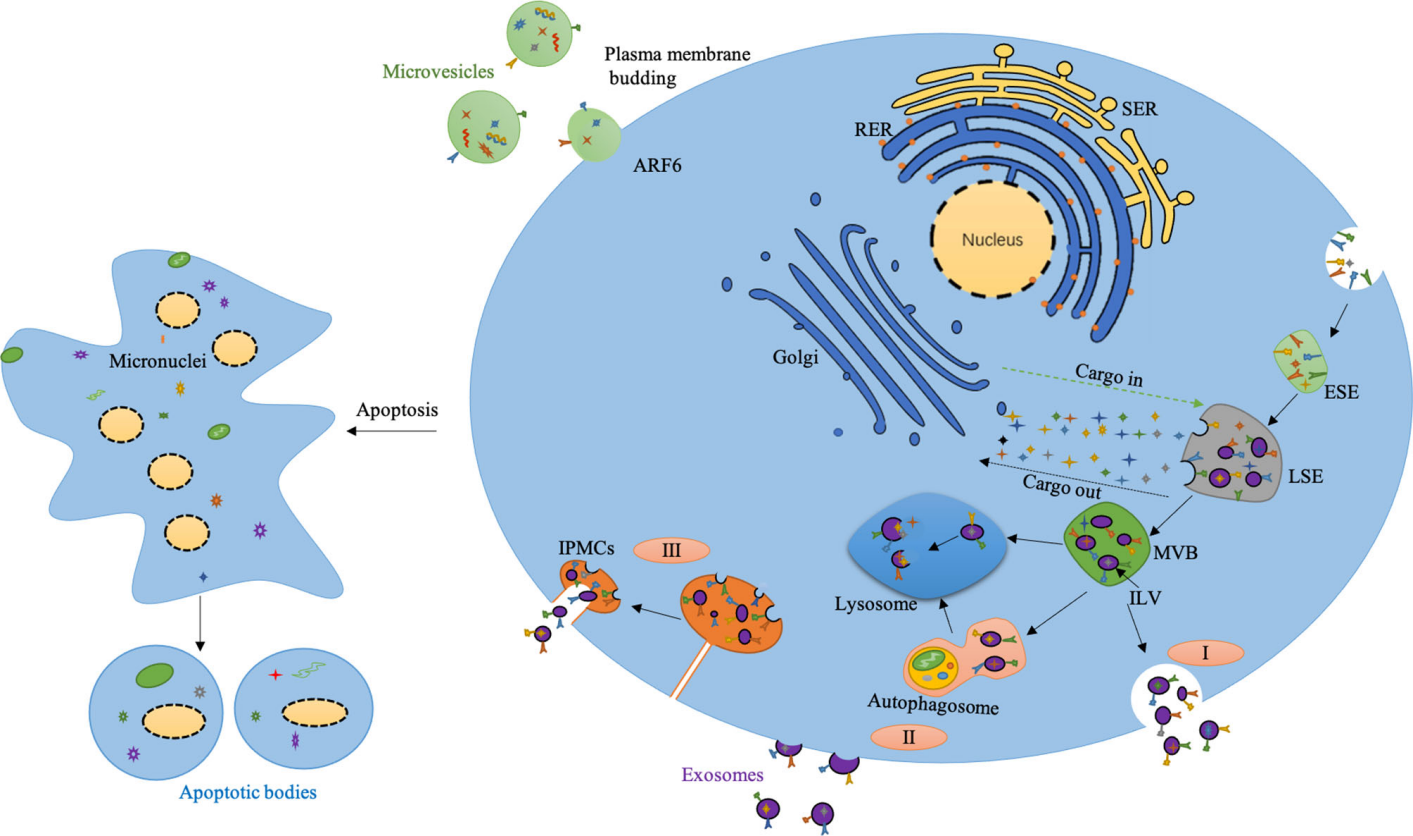
Biogenesis of extracellular vesicles. I) The classical pathway of exosomes biogenesis. II) Exosomes are released directly from the plasma membrane by budding. III) Exosomes released by budding at intracellular plasma membrane–connected compartments (IPMCS). The neck channel of the IPMCS becomes wider, the neck restraint is released, and the exosomes are released outside. Micro-vesicles are produced by plasma membrane budding mediated by ARF6. Apoptotic bodies are produced from apoptotic cells. SER, Smooth endoplasmic reticulum; RER, Rough endoplasmic reticulum; ESE, Early sorting endosome; LSE, Late sorting endosome; MVB, Multivesicular body; ILV, Intraluminal vesicle.

Currently, the classical pathway of exosomes is generally well-known. Specifically, the plasma membrane first buds inward through endocytosis to form small cup morphology, which contains some proteins on the cell surface and partial substances in the extracellular environment. These structures are namely early-sorting endosomes (ESEs). The Golgi apparatus and endoplasmic reticulum also participate in ESEs’ formation, resulting in late sorting endosomes (LSEs). LSEs eventually lead to the formation of multivesicular bodies(MVBs). MVBs could subsequentially fuse with autophagosomes and lysosomes, and finally get degraded through this way. Alternatively, they can fuse directly with lysosomes and get degraded. They can also fuse with the plasma membrane and release various ILVs in the form of exosomes (Hessvik and Llorente, 2018; Kalluri and LeBleu, 2020). MVBs play an important role in the classification, recovery, transportation, storage and release of proteins in the classical pathway (Borges et al., 2013). Beyond MVBs-mediated biogenesis of exosomes, the cells could directly produce exosomes by budding from plasma membrane, which has been evidenced by electron microscope. Exosomes could also be derived from intracellular plasma membrane–connected compartments (IPMCs). The neck channel of the IPMCs initially turns wider with following release of the neck restraint (**Figure 1**). IPMCs also overlap with the budding site of retroviruses, emphasizing the similarity between exosomes and virus. These two mechanisms are also supported by the germination process of CD9 and CD63 proteins (van Dongen et al., 2016; Pegtel and Gould, 2019).

The production of exosomes is also tightly regulated. Munc13-4 is a Ca^2+^-dependent soluble NSF attachment protein (SNAP) receptor and Rab-binding protein, which is necessary for Ca^2+^-dependent membrane fusion. The combination of Munc13-4 and Rab11 can mediate the production of MVBs, and the interaction between Munc13-4 and Ca^2+^ also provides a new way for the increase of intracellular Ca^2+^ to promote exosomes’ production (Messenger et al., 2018). Studies have also shown that the final destination of a particular MVB depends in part on cholesterol levels in MVBs. Specifically, cholesterol-rich vesicles are secreted, while cholesterol-deficient vesicles with the same shape are sent to lysosomes for further degradation (Mobius et al., 2002). Besides, the stress signal transduction molecules inositol required enzyme 1 (IRE1) and PKR-like ER kinase (PERK) on the endoplasmic reticulum (ER) mediate the formation of ER stress-dependent MVBs and exosomes. IRE1 and PERK mediate the phosphorylation regulation of molecules related to MVBs formation, which also provides a novel mechanism for ER stress tolerance of autophagy clearance response (Kanemoto et al., 2016). The PH value in the cells also affects the generation of exosomes. Studies have shown that acidic conditions promote the production of exosomes, while alkaline conditions inhibit this process (Parolini et al., 2009; Ban et al., 2015). Moreover, hypoxia is another critical element which regulates the production of exosomes. Once tumor cells exposed to hypoxia, the production of exosomes increased due to upregulated hypoxia-inducible factor-1α (HIF-1α). Other factors such as endosomal sorting complexes required for transport (ESCRT), lipids, syndecan, syntenin, Rab GTPases, SNAP, cytoskeleton also play an important role during the formation and secretion of exosomes (Mathieu et al., 2019; Kalluri and LeBleu, 2020). Collectively, due to diverse regulation mechanism, the size, contents and types of exosomes are highly heterogeneous.

### Biogenesis of Micro-Vesicles

The production of micro-vesicles is mainly through the budding of plasma membrane (PM). The formation of micro-vesicles results from dynamic interactions between phospholipid redistribution and cytoskeletal proteins contraction. The outer layer of PM mainly contains sphingomyelin and phosphatidylcholine while the inner layer is composed of phosphatidylethanolamine and phosphatidylserine. This asymmetric distribution ensures the “laterality” of membrane lipids. However, the influx of Ca^2+^ into the cytoplasm breaks this lipid asymmetry by activating the scramblase that promotes lipids turnover and remixing. This activation reaction in turn leads to lipids redistribution on the cell membrane, promoting cells membrane blistering (Hugel et al., 2005; Akers et al., 2013). A current study revealed ARF 6-GTP dependent on Phospholipase D activates ERK recruitment to the PM. ERK phosphorylates and activates myosin light-chain kinase (MLCK), which is a Ca^2+^ dependent protease. MLCK activation could promote MLC phosphorylation, and this will allow the neck of the microcapsule to contract based on actomyosin and promote the release of the microcapsule into the extracellular space. Micro-vesicles derived from specific sites of the cell transport some featured cellular components, such as extracellular matrix (ECM) and cell adhesion. ARF6 could regulate the shedding of micro-vesicles in tumor cells, while the relevant integrin receptors on the surface of the micro-vesicles could bind the extracellular matrix (ECM). Furthermore, micro-vesicles contain some enzymes, which can perform degradation functions in specific parts of ECM, thereby destroying the stability of ECM and promoting tumor metastasis (Muralidharan-Chari et al., 2009).

### Biogenesis of Apoptotic Bodies

Apoptotic bodies are small vesicles containing part of both nuclear and cytoplasm fragments released by cells under the process of programmed death (Quan et al., 2021). The production of apoptotic bodies mainly undergoes three processes. First, the nuclear chromatin concentrates, then the cell membrane blisters, and finally the cell contents disintegrate to form vesicles wrapped by membrane, namely apoptotic bodies (Akers et al., 2013). The formation of apoptotic bodies is a physical process caused by the increase of hydrostatic pressure after cells contraction (Borges et al., 2013). Phagocytes can recognize apoptotic bodies and mediate clearance responses (Croft et al., 2005). Apoptotic bodies can interact with recipient cells through cell surface interactions, membrane fusion, endocytosis (Mulcahy et al., 2014; Xu et al., 2019). Compared to exosomes and micro-vesicles, apoptotic bodies are mainly produced by apoptotic cells. Studies have shown that some of the biologically active substances in apoptotic bodies can stimulate the collective immune system, replenish dead cells, and mediate pathophysiological damage and repair. Their important biological functions provide the possibility to develop new treatment strategy (Quan et al., 2021). Based on what has been discussed above, apoptosis bodies are heavily responsive to stress especially apoptosis. However, the contents, number and size of exosomes, micro-vesicles may vary depends on many kinds of stress stimuli. Here we include exosomes, micro-vesicles and apoptotic bodies collectively as EVs.

## The Role of EV contents in Sepsis and ARDs

The pathogenesis of sepsis is extremely complex on the cellular and molecular levels, such as imbalance in inflammatory response, immune dysfunction, mitochondrial damage, coagulopathy, neuroendocrine immune network abnormalities, endoplasmic reticulum stress, autophagy, and other pathophysiological processes, and ultimately leads to organ dysfunction. The pathogens that induce the diseases are mainly bacteria, fungi, parasites and viruses (Huang et al., 2019). Sepsis-mediated lung damage eventually leads to ARDS. Therefore, early prevention of pathogens infection can effectively reduce the incidence of sepsis and ARDS. Pathogens can interact with host cells in a variety of ways. EVs may play a key role in the interaction between pathogens and host cells during infection (Schorey et al., 2015). The role of EVs in Sepsis and ARDS mainly depends on nucleic acids, lipids, proteins and other components. EVs represent a unique mode of intercellular communication by serving as vehicles for transfer of membrane proteins, extracellular matrix proteins, nuclear proteins, metabolites and nucleic acids (Raposo and Stoorvogel, 2013). These molecular substances transferred by EVs can modify the function of recipient cells and participate in a variety of biological processes, including blood vessel growth, immune response, tumor migration and invasion (Driedonks and Nolte-'t Hoen, 2018; Xu et al., 2018).

### The Role of EVs Non-Coding RNAs in Sepsis and ARDS

RNAs detected in EVs consist mRNA, LncRNA, circRNA, miRNA, tRNA, rRNA, snoRNA, snRNA, piRNA, Y-RNA, SRP-RNA (7SL), and Vault RNA (Kim et al., 2017; Driedonks and Nolte-'t Hoen, 2018). Non-coding RNAs have been demonstrated to participate in various biological processes, including apoptosis, mitochondrial dysfunction, and innate immunity (**Table 1**) (Hashemian et al., 2020). Accumulating data shows EVs-derived non-coding RNAs differentially expressed in the body fluids of patients with sepsis and sepsis-induced ARDS compared with healthy donors, playing fundamental role in occurrence and progression of the disease by regulating cell activities (Chen et al., 2020).

**Table 1:** The EVs-derived non-coding RNAs in sepsis and ARDS.

Effect on disease	EVs source	non-coding RNAs in EVs	Target cell types or genes	Biological function	References
Anti-inflammatory response	Macrophage	miR-223	TLR4/NF-κB	negatively regulate the TLR4/NF-KB pathway in macrophages and NLRP3 inflammasome activation	(Wang et al., 2015; Yan et al., 2019)
	Macrophage	miR-142	NLRP3 inflammasome	suppress NLRP3 inflammasome activation and bacterial infection-associated lung inflammation	(Zhang D. et al., 2019)
	MSCs	miR-146a		Induces macrophages to polarize to M2 type, thereby exerting the anti-inflammatory effect of M2 type macrophages	(Song et al., 2017)
	MSCs	miR-21		Inhibit the effect of PCD4, induce macrophages to polarize to M2 type, thereby exerting the anti-inflammatory effect of M2 type macrophages	(Yao et al., 2021)
	MSCs	miR-27a-3p		Promote the transformation of BMDM to M2 type, inhibit the expression of KB subunit 1 of alveolar macrophages, thereby inhibiting the inflammatory response mediated by the NF-KB inflammatory signal pathway	(Wang et al., 2020)
	MDSCs	Lnc-Hotairm1		inhibit both T cell proliferation and IFN-ϒ production	(Alkhateeb et al., 2020)
	EPCs	Lnc-TUG1	MiR-9-5p	promote M2 macrophage polarization and ameliorate sepsis-induced organ damage	(Ma et al., 2021)
	Neutrophils	miR -150 miR-451a		Induce anti-inflammatory macrophage	(Youn et al., 2021)
	EPCs	miR-126	HMGB1,VCAM1 SPRE D1,RAF/ERK1 caspase-3/9 PIK3R2 VEGF-α	Increase epithelial tight junction protein expression while decreasing target genes with relevance to ALI	(Wu et al., 2018; Zhou et al., 2019; Fujimoto et al., 2020; Zou et al., 2020)
Pro-inflammatory response	Neutrophils	miR -1260 miR -1258 miR -4454 miR -7975		Promote pro-inflammatory macrophage polarization	(Youn et al., 2021)
	peripheral blood	miR -155	SHIP1 SOCS1	promote M1 macrophage proliferation and inflammation	(Balusu et al., 2016; Jiang et al., 2019)
	MSCs	Lnc-p21	miR-181 SIRT1	suppress epithelial cells apoptosis and alleviate lung tissue injury	(Sui et al., 2021)
	BMDMs	miR-466 family	NLRP3 inflammasome	Activate NLRP3 inflammasome and exacerbate inflammation	(Shikano et al., 2019)

### EVs-miRNAs

Current studies on the role of EVs-derived non-coding RNAs in sepsis field are mainly focused on miRNAs. The miRNAs are small, endogenous, non-coding RNAs of 22 to 26 nucleotides in length that function primarily as post-transcriptional regulators of gene expression (Essandoh et al., 2016). EVs-miRNAs have been demonstrated to be associated with inflammatory conditions, metabolic disorders, malignancies, and autoimmune disorders (Fujimoto et al., 2020). Numerous studies have reported that miRNAs are capable of regulating inflammatory responses *via* targeting the Toll-like receptor (TLR) signaling pathway, which plays a crucial role in sepsis. TLRs activate NF-kB and stimulate innate immunity and inflammatory responses through interleukin 1 (IL-1) receptor-like pathway (Williams et al., 2003). For instance, miRNA-223 negatively regulates the TLR4/NF-kB pathway in macrophages and the activation of nucleotide-binding oligomerization like receptor 3 (NLRP3) inflammasome, attenuating lipopolysaccharide (LPS)−induced inflammation in ALI (Wang et al., 2015; Yan et al., 2019). Serum EVs-containing miR-155 from ALI mice could stimulate NF-kB activation and further induce the production of tumor necrosis factor-α (TNF-α) and IL-6, thereby promoting LPS-induced lung injury (Jiang et al., 2019). MiRNA-142 from macrophage-derived EVs could also suppress NLRP3 inflammasome activation and bacterial infection-associated lung inflammation (Zhang D. et al., 2019). The miRNA-466g and miRNA-466m in bronchoalveolar lavage fluid (BALF) could exacerbate inflammation in ARDS mouse model as precipitating factors for the NLRP3 inflammasome. Therefore, it could be speculated that the miR-466 family, miRNA-223 and miRNA-124 might be the effector molecules involved in EVs’ regulation of NLRP3 (Shikano et al., 2019). However, the specific source of EVs in BALF has not yet been clearly clarified. Macrophages play a critical role in the pathogenesis of lung inflammation as essential effector cells of host defense against foreign stimuli (Jiang et al., 2019). Several kinds of miRNAs have been identified to modulate macrophage proliferation. IL-1β induces a significant miRNA-146a level package into EVs from mesenchymal stromal cells (MSCs), which induced macrophages to M2 macrophages polarization by regulating signals such as IRAK1, TRAF6, and IRF5, thereby exerting the anti-inflammatory effect of M2 macrophages, and ultimately play a protective role in sepsis (Song et al., 2017). Moreover, the significant up-regulation of miR-21 in MSC-EVs caused by IL-1β can induce the M2 polarization of macrophages by inhibiting the effect of programmed cell death 4 (PDCD4), with similar pattern as miRNA-146a in sepsis (Yao et al., 2021). Studies have shown that miR-27a-3p in EVs secreted by MSCs can promote the transformation of bone marrow-derived macrophages (BMDM) to M2 type, and inhibit the expression of alveolar macrophages NF-kB subunit 1, thereby inhibiting the NF-kB signaling pathway and alleviating the progression of ARDS (Wang et al., 2020). Serum EVs-derived miR-155 could promote M1 macrophage proliferation and inflammation by targeting SH2 domain-containing inositol 5’-phosphatase 1 (SHIP1) and suppressor of cytokine signaling 1 (SOCS1) (Jiang et al., 2019).

In addition, miRNA-126 delivered by endothelial progenitor cells (EPC)-derived EVs can increase epithelial tight junction proteins expression but decrease target genes with relevance to ALI such as phosphoinositide-3-kinase regulatory subunit 2 (PIK3R2), high mobility group box 1 (HMGB1), vascular cell adhesion molecular1 (VCAM1), and vascular endothelial growth factor-α (VEGF-α), thereby reducing lung microvascular endothelial inflammation and repairing injured endothelial cells (Zhou et al., 2019; Fujimoto et al., 2020). Neutrophil-derived micro-vesicles (NDMVs) and neutrophil-derived trails (NDTRs) are two subtypes of neutrophil-derived EVs. NDTRs contain proinflammatory miRNAs such as miR-1260, miR-1285, miR-4454 and miR-7975, while NDMVs carry anti-inflammatory miRNAs including miR-126, miR-150, and miR-451a. NDTRs promote pro-inflammatory macrophage polarization whereas NDMVs induce anti-inflammatory macrophage polarization (Youn et al., 2021). Balusu reported that the amount of EVs in cerebrospinal fluid (CSF) increased when systemic inflammation occurred (Balusu et al., 2016). And the miRNAs-containing EVs derived from choroid plexus epithelium (CPE) cross the ependymal cell layer lining the ventricles, reach the recipient brain parenchymal cells, and induce the repression of target mRNAs and inflammatory response activation. Besides, EVs-derived miRNAs that play a role in inflammation include miR-155 and miR-146 (Balusu et al., 2016).

### EVs-LncRNAs

In the regulation of inflammatory response, LncRNAs usually exert “sponge-like” effects on various miRNAs or act as a competing endogenous RNA (CeRNA) to affect the expression and function of miRNAs, thereby indirectly modulating the inflammatory response (Bayoumi et al., 2016). For example, Lnc-MALAT1 could sponge miR-149 (Liang et al., 2019), miR-146a (Dai et al., 2018) and miR-425 (Wang et al., 2019) and Lnc-PRNCR1 could sponge miR-330-5p (Yu et al., 2021). Lnc-SNHG5 could sponge miR-205 (Wang J. et al., 2021) in LPS-induced ARDS model and Lnc-XIST can sponge miR-146a-5p (Li J. et al., 2021). Lnc-TUG1 transmitted by EPC-derived EVs could bind to miR-9-5p, promote M2 macrophage polarization and ameliorate sepsis-induced organ damage (Ma et al., 2021). LncRNA-p21 from MSC-EVs could downregulate miR-181, thus suppressing epithelial cells apoptosis and alleviating lung tissue injury by upregulating the expression of sirtuin1(SIRT1) (Sui et al., 2021). These “sponge-like” effects contribute to the pathogenesis of systematic inflammation and are expected to provide ideas for diagnosis and treatment of sepsis and sepsis-induced ARDS.

Immunosuppression usually occurs in late stage of sepsis, such as suppression of T cell activation and proliferation. EVs from late sepsis Gr1^+^CD11b^+^ cells significantly inhibit both T cell proliferation and Interferon ϒ (IFN-ϒ) production, and this effect might be accomplished by the high level of LncRNA-Hotairm1 in myeloid-derived suppressor cells (MDSCs)-derived EVs (Alkhateeb et al., 2020). EVs-associated Y-RNA from immune cells may be involved in a range of immune-related processes such as inflammation and immune suppression (Driedonks and Nolte-'t Hoen, 2018). So far, the role of EVs-derived non-coding RNAs in the immune system of sepsis and ARDS is still not clear enough, more underling mechanisms are still yet to get revealed.

### The Role of EVs Proteins in Sepsis and ARDS

#### EVs-Proteins in Sepsis

The alpha-2-macroglobulin (A2MG) in EVs promoted the adhesion of neutrophils to endothelial cells in humans, enhanced the phagocytosis of human macrophages, and preserved the chemotaxis of neutrophils in the presence of LPS (**Table 2**). Furthermore, The A2MG in EVs also protected sepsis mice from hypothermia, reduced bacterial titers, increased levels of immune-decomposing lipid mediators in inflammatory exudates, and reduced systemic inflammation. A2MG also improved survival in mice with sepsis (Dalli et al., 2014). It happens that there is a similar case, milk fat globule epidermal growth factor-factor VIII (MFG-E8) has also been found with protective effect on sepsis. In sepsis, apoptotic cells would cause host damage once not timely cleared by specialized macrophages. Integrin αvβ3, bridging protein and MFG-E8 play an important role in the complete removal of these apoptotic cells. By setting up control experiments, it was found that MFG-E8 contained in EVs secreted by immature dendritic cells (IDCs) can reduce the level of inflammation in patients with sepsis and reduce the symptoms of sepsis by enhancing the ability of macrophages to remove apoptotic cells. There may be many sources of IDCs and our research found treatment with bone marrow dendritic cell-derived EVs can also reduce plasma tumor necrosis factor α (TNF-α) and IL-6 levels and improve survival rate. Therefore, the active elimination of apoptotic cells by macrophages may provide a new anti-sepsis treatment method to prevent the development of secondary necrosis (Miksa et al., 2006; Miksa et al., 2009). Not only can MFG-E8 reduce the release of pro-inflammatory factors, heat-shock protein 12B (HSPA12B) can also work in similar pattern. HSPA12B family encapsulated by EVs secreted by endothelial cells could lead to down-regulation of the activation of NF-kB and nuclear translocation in LPS-stimulated macrophages. It reduces the level of inflammatory factors such as TNF-α and IL-1β produced by macrophages, and increases the level of macrophages derived anti-inflammatory factor IL-10, functioning protective role in sepsis (Tu et al., 2020). The HSP70 protein family has strong functions, which can not only inhibit the occurrence of inflammatory responses, such as HSPA12B mentioned above, but also promote the occurrence of inflammatory responses, and thus promote sepsis. For example, EVs produced by macrophages infected with mycobacteria containing another member of the HSP70 family exerts a pro-inflammatory effect by activating the NF-kB inflammatory pathway in uninfected macrophages and stimulating the release of TNF-α (Anand et al., 2010).

**Table 2:** The proteins of EVs in sepsis and ARDS.

Effect on disease	EVs source	Proteins in EVs	Target cell types or genes	Biological function	References
Anti-inflammatory response	Using energy-induced conversion to neutrophil’ EVs to make the A2MG-enriched EVs	A2MG	LRP1 receptor	Promote the adhesion of neutrophils and endothelial cells, enhance the phagocytosis of macrophages, maintain the chemotaxis of neutrophils in the presence of LPS, reduces bacterial titers, and reduces systemic inflammation.	(Dalli et al., 2014)
	IDCs	MFG-E8	Macrophages	Enhance the phagocytes to engulf apoptotic cells by interaction with integrin αvβ3	(Miksa et al., 2006; Miksa et al., 2009)
	Endothelial cells	HSPA12B	NF-KB	Suppression of activation and nuclear translocation of NF-KB, reduces the level of inflammatory factors	(Tu et al., 2020)
	MPMVECs	Syndecan-1	FAK/p190RhoGAP/RhoA/ROCK/NF – KB signaling pathways	Reduce the release of pro-inflammatory factors caused by LPS, protects glycocalyx, reduces the number of cells and protein levels in BALF	(Zhang C. et al., 2019)
	MSCs	HGF	endothelial cells	Increase the expression of VE-cadherin and occludin between endothelial cells, induce endothelial proliferation, reduce endothelial cells apoptosis, and stabilize the endothelial barrier. HGF can also inhibit the expression of IL-6 pro-inflammatory factors in endothelial cells and promote IL-10 anti-inflammatory factors Expression	(Mason, 2002; Wang et al., 2017)
Pro-inflammatory response	Fungi (such as Cryptococcus neoformans)	Laccase	macrophages	The decrease in laccase-containing EVs secreted by fungi results in decreased synthesis of melanin, which in turn reduces the virulence of fungi (inhibiting the phagocytosis of macrophages), and thus plays a protective role in Sepsis	(Eisenman et al., 2009; Panepinto et al., 2009; Liu et al., 2014)
	Macrophages infected with Mycobacterium avium	TLR ligand	Uninfected macrophages TLR2, TLR4 and MyD88 receptor	EVs secreted by Mycobacterium avium-infected macrophages can cause inflammation in surrounding uninfected macrophages, thereby promoting the occurrence of sepsis	(Bhatnagar and Schorey, 2007)
	Macrophages infected with mycobacteria	A HSP70 protein	Activate NF-KB activation and TNF-α release in uninfected macrophages	Activate the inflammatory pathways, promote the release of pro-inflammatory factors, increase the inflammatory responses and advancing sepsis	(Anand et al., 2010)
	BMDM	Histones	The TRL-4 receptor	Promote the release of pro-inflammatory factors TNF-α, IL-6, IL-1β, the occurrence of inflammatory response mediates organ damage, increase the mortality of sepsis.	(Xu et al., 2009; Nair et al., 2018; Burgelman et al., 2021; Li Y. et al., 2021)
	Macrophages	HMGB1	RAGE, Toll-like receptor TLR2, TLR4	bind to the receptors of a variety of pro-inflammatory pathways, exert its pro-inflammatory effects	(Tsung et al., 2014; Chen et al., 2016)
	Sepsis patients’ plasma	CRP	Activate complement and macrophage	stimulate the release of IL-8 from cultured human monocytes, trigger an inflammatory response	(Fendl et al., 2021)
	Lung	IL-1β/IL-18	P2X7/caspase-1	increase the accumulation of neutrophils, exerts a pro-inflammatory response, leading to aggravation of ARDS	(Eltom et al., 2014)
	PNM	NE	ECM	Integrin MAC-1 on EVs can bind to ECM and mediates NE in EVs to degrade ECM, causing disorders of ECM homeostasis	(Genschmer et al., 2019)

The EVs from sepsis patients may also contain histones, which are actually nuclear proteins that pack the nuclear DNA into chromatin (Marino-Ramirez et al., 2005; Burgelman et al., 2021). The EVs containing histones released by mouse-derived BMDM interact with TLR4, displaying pro-inflammatory effect, which can promote the production of TNF, IL-6 and IL-1β, thereby increasing the lethality of sepsis (Xu et al., 2009; Nair et al., 2018; Burgelman et al., 2021; Li Y. et al., 2021). HMGB1 represents another type of protein displaying pro-inflammatory effect in sepsis disease. HMGB1 can be passively released when stimulated by pressure. But when immune cells are activated, HMGB1 can be actively released into EVs. Tobacco smoke extract (TSE) can induce the release of EVs containing HMGB1 from macrophages, which can bind to receptors in a variety of pro-inflammatory pathways (such as receptor for advanced glycation end products, TLR2, TLR4), and then exert the HMGB1 pro-inflammatory effect, promoting the occurrence of sepsis (Tsung et al., 2014; Chen et al., 2016). C-reactive protein (CRP) also plays an irreplaceable role in the pathogenesis of Sepsis. The level of CRP in plasma EVs of sepsis patients was significantly higher than that of the control group. CRP can change its own structure to stimulate the release of IL-8 inflammatory factors from cultured human monocytes, which can further trigger an inflammatory response. And CRP in the plasma circulation is conducive to the occurrence of systemic inflammatory responses (Fendl et al., 2021). Laccase is also a common and indispensable factor in the responses of sepsis and ARDS caused by fungal infection. Studies have shown that by knocking down the Sec6 gene involved in EVs secretion, the release of EVs containing laccase in the fungi is reduced, thereby reducing the fungal virulence (Panepinto et al., 2009). Laccase is an enzyme necessary for the synthesis of melanin, which is an important molecule for fungal pathogenicity (Eisenman et al., 2009). The mechanism of melanin in fungal pathogenicity is that melanin on the fungal cell wall can inhibit the phagocytosis of macrophages and promote the occurrence of fungal diseases (Liu et al., 2014).

In addition, during pathogens infection, EVs released by host cells taste pathogen-containing components that may mediate interactions with host cells. The process of infection of macrophages by Mycobacterium avium is a good example. The EVs secreted by Mycobacterium avium-infected macrophages can promote the inflammatory response of the surrounding uninfected macrophages. This pro-inflammatory response depends on TLR2, TLR4 and MyD88, suggesting that EVs released by macrophages contain TLR ligands expressed by Mycobacterium avium (Bhatnagar and Schorey, 2007). In addition, the surface of EVs can also bind various cytokines such as IL-1β, IL-18, GRO-α, IP-10, M-CSF, MCP-1, etc. These EVs surface cytokines are able to increase local cytokine levels, which have important implications regarding inflammatory processes (Fitzgerald et al., 2018; Burgelman et al., 2021). ARDS is a severe hypoxic respiratory failure caused by pneumonia, sepsis, trauma and other risk factors. Among them, sepsis is considered to be the main cause of ARDS. It is characterized by diffuse alveolar damage (DAD), apoptosis of alveolar type I and II cells, destruction of alveolar capillary barrier, accumulation of protein-like edema in the alveolar cavity and increase of inflammatory cells (Thompson et al., 2017; Zhou et al., 2019).

#### EVs-Proteins in ARDS

The proteins in EVs not only play a key role in sepsis, but also play an important role in the occurrence and development of ARDS. Numerous studies were focused on EVs secreted by infected mouse pulmonary microvascular endothelial cells (MPMVECs) with employing LPS-induced ALI mice. EVs up-regulation by syndecan-1 could alleviate pulmonary edema and inflammation, reduce BALF proliferation and relative intracellular proteins level, and protect glycocalyx. In addition, the up-regulation of syndecan-1 in EVs also reduced the expression of LPS-induced proinflammatory cytokines IL-1β, TNF-α and IL-6. It can also reduce the formation of stress fibers after LPS stimulation and improve the high permeability of monolayer. Studies have shown that EVs containing high levels of syndecan-1 can exert anti-inflammatory effects through the FAK/p190RhoGAP/RhoA/ROCK/NF-kB signaling pathway, and protect organs, thereby exerting a protective effect in the occurrence and development of ARDS (Zhang C. et al., 2019). Hepatocyte growth factor (HGF) can also play an important role in inhibiting the development of ARDS. HGF mediates the epithelial-interstitial interaction of lung regeneration after lung injury. The results showed that HGF in EVs secreted by MSCs acted on the endothelial intercellular junction proteins VE-cadherin and occludin. HGF in EVs secreted by MSCs can also reduce the apoptosis of endothelial cells, induce the proliferation of endothelial cells, and protect and stabilize the endothelial barrier function. HGF in EVs can also inhibit the expression of IL-6 pro-inflammatory factors in endothelial cells and promote the expression of IL-10 anti-inflammatory factors. This experiment indicates that HGF in EVs secreted by MSCs can partially inhibit the occurrence of ALI, and thus preventing the occurrence of ARDS (Mason, 2002; Wang et al., 2017).

ATP can also act as an agonist of EVs, thereby mediating the role of EVs in ARDS. When bacteria and viruses cause respiratory infections, they can trigger the release of functional EVs in mice and humans, and the ATP levels in the lungs of Asthma/chronic obstructive pulmonary disease (COPD) patients are increased. Once stimulated by ATP, EVs release IL-1β and IL-18 in a P2X7/caspase-1 axis dependent manner, resulting in exacerbated neutrophilia. Inflammatory factors can activate inflammatory signal pathways to exert their pro-inflammatory effects, which in turn exacerbate Asthma/COPD symptoms (Eltom et al., 2014). Similar to the above, the NE and integrin MAC-1 molecules in EVs can also aggravate the occurrence and development of COPD. In COPD, activated neutrophils (PNMs) release CD63+/CD66b+ EVs carrying neutrophil elastase (NE). The integrin MAC-1 on EVs can bind to ECM and mediates NE in EVs to degrade ECM, causing disorders of ECM and resulting in the aggravation of ARDS, COPD, bronchopulmonary dysplasia, BPD) and provide a key mechanism for proteolytic damage (Genschmer et al., 2019). Other studies also have found that secretory phospholipase-IIA A2 (sPLA2-IIA) is related to the occurrence of ALI/ARDS. The results show that sPLA2-IIA can be used as an early diagnostic indicator of ARDS. In the lung injury caused by LPS, it can hydrolyze the lung surfactant phospholipid, destroy the stability of the lung surfactant (LS) monolayer, and then directly deteriorate the lung function, ultimately promoting the occurrence and development of ARDS (Arbibe et al., 1997; Kitsiouli et al., 2009; Spyridakis et al., 2010; Papadopoulos et al., 2020). Finally, in the process of BCG and Mycobacterium infection, some proteins produced by host cells also have the effect of pro-inflammatory response. For instance, EVs isolated from the BALF of BCG-infected mice contain mycobacterial components lipoarabinomannan and 19-kDa lipoprotein, which can stimulate naive macrophages to produce TNF-α. EVs isolated from macrophages infected with BCG and Mycobacterium tuberculosis could stimulate TNF-α and IL-12, as well as neutrophils and macrophages to recruit in the lungs. And then play a role in promoting the occurrence and development of ARDS (Bhatnagar et al., 2007). EVs proteins function as a double-edged sword, which can play a protective role or aggravate injury. The exact regulation mechanism of EVs secretion among different stages of sepsis is largely related to the parent cells’ state. For example, in the early stage of inflammation imbalance, the invasion of some pathogens may induce macrophages to secrete and release some pro-inflammatory EVs, delivering corresponding pro-inflammatory signals, thus promoting the occurrence of inflammatory response. However, during the injury repair stage of the disease, some signals may stimulate macrophages, thus promoting the M2-type polarization of macrophages and thereby playing their anti-inflammatory activity. The exact mechanism underlying time-sensitive for EVs secretion still require more effective and leading-edge techniques to further analyze and verify. In addition, certain types of proteins in EVs may be serving as potential therapeutic targets and diagnostic indicators in sepsis (Kronstadt et al., 2021).

### The Role of EVs Metabolites in Sepsis/ARDS

Uncontrolled activation of the coagulation cascade is common in sepsis and is an important cause of the hypercoagulable state of death associated with disseminated intravascular coagulation (DIC) (Semeraro et al., 2012). Although tissue factor (TF) plays a key role in clotting activation in sepsis and blocking TF activity is theoretically considered the most reasonable treatment, its use as a therapeutic target in the treatment of sepsis is controversial (Levi et al., 2013; King et al., 2014). Studies have shown that PS exposed on EVs surface can play a good coagulation effect in sepsis, so the development of new target drugs for PS provides new ideas for the treatment of sepsis (Zhang et al., 2016).

## Clinical Application of EV in Sepsis/ARDS

### EVs as Diagnostic Biomarkers

In clinical, for patients with infection or suspected infection, sepsis can be diagnosed when the sequential (sepsis-related) organ failure assessment (SOFA) score is greater than or equal to 2 points from the baseline. As for the diagnostic criteria of ARDS, the Berling definition elaborated ARDS diagnosis requires that new or worsening respiratory distress and bilateral chest radiographical abnormalities be present for 7 days or fewer, that heart failure cannot fully explain the hypoxemia and radiographical infiltrates, and that the impaired oxygenation be clinically significant. (Meyer et al., 2021) The current diagnostic criteria for sepsis and ARDS are based on clinical symptoms. However, biomarkers also play non-ignorably important roles in judging and predicting disease development.

**Figure 2:**
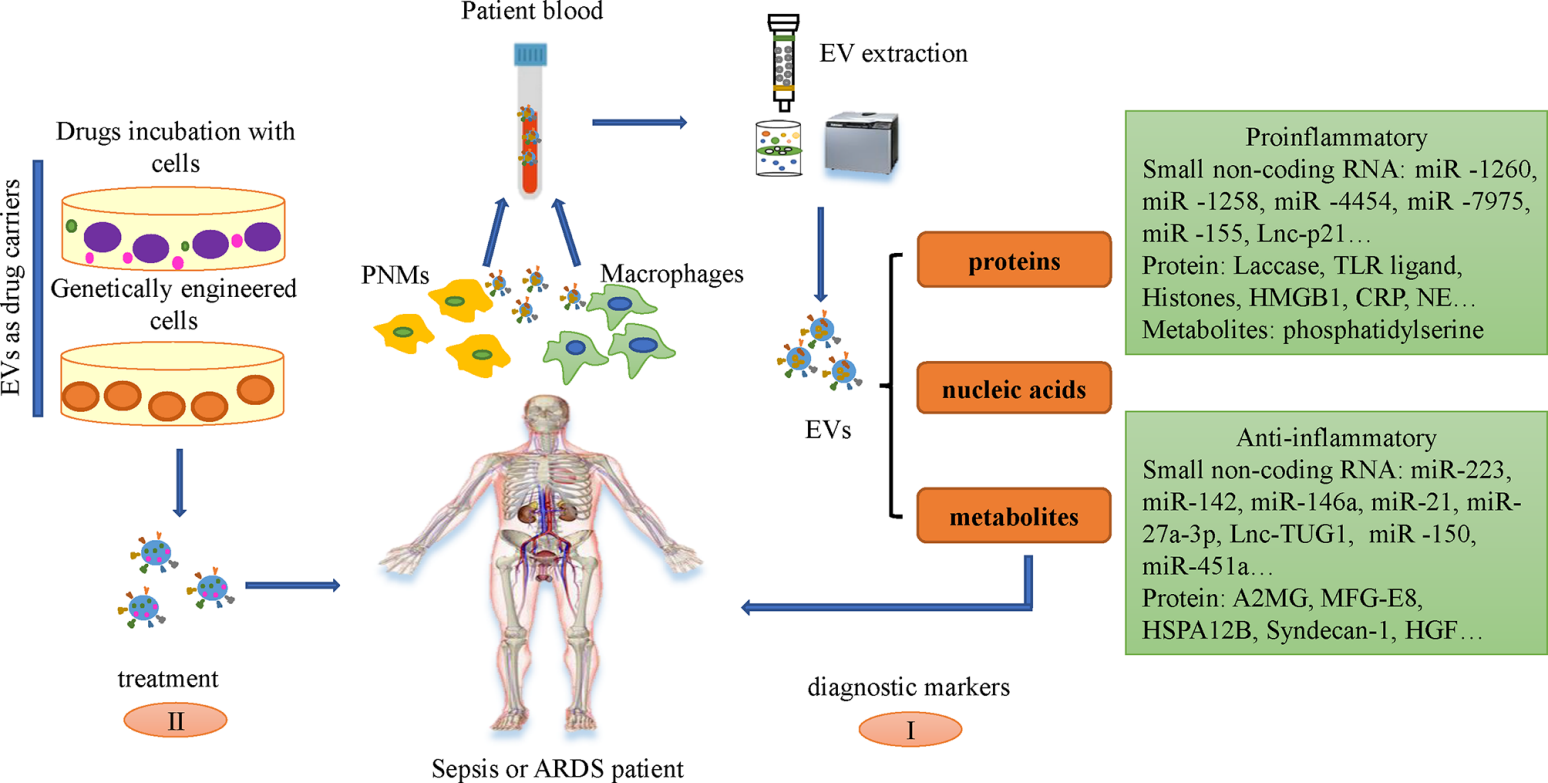
Application of EVs in sepsis and ARDS. I. Small non-coding RNAs and proteins in the serum EVs of patients with sepsis and ARDS can be used as diagnostic markers. II. EVs could be engineered and employed as drug delivery vehicles for downstream treatment *via* two different ways: incubate the drugs with the cells to produce drugs-loaded EVs and engineer the genetic characteristics of cells through genetic remodeling, making EVs with designed bio-active cargoes.

Biomarkers refer to biochemical indicators that can mark the changes or possible changes in the structure or function of systems, organs, tissues, cells and sub-cells, through which we can know the current biological process of the body. Bacterial culture is the golden standard for differential diagnosis of sepsis, but it is often time-consuming with low accuracy. So the search for new diagnostic markers during sepsis remains meaningful and needed (Wang C. et al., 2021). L-lactate, CRP, and procalcitonin (PCT) are serving as specific biomarkers in sepsis. Other available biomarkers include cytokines and chemokine markers, such as IL-6, IL-10, IL-8, and cell surface markers and soluble receptors, including HLA-DR, CD64, soluble triggering receptor expressed on myeloid cells-1 (sTREM-1), soluble urokinase plasminogen activator receptor (suPAR). In addition, vascular endothelial cells related markers, such as cells adhesion molecule, angiopoietin, endocan, and coagulation related markers, including PAI-1, antithrombin III, also contribute significant information regarding sepsis diagnosis, severity and prognosis. However, due to the complexity of sepsis, an early, accurate and reliable biological markers that can be used as the “gold standard” for the diagnosis and prognosis of sepsis is still unavailable. One study demonstrated the amount of plasma EVs in sepsis patients was associated with organ failure and mortality (Hubert et al., 2015; Kubo, 2018; Im et al., 2020). The profiles of comprised miRNAs are associated with the progression of sepsis (Hashemian et al., 2020). MiR-21-5p, miR-30a-5p, miR-192-5p, miR-26a-5p, miR-23a-5p, miR-191-5p and miR-193a-5p are differentially expressed in EVs between healthy volunteers and septic patients and could be identified as potential early indicators for sepsis and septic shock (**Figure 2**) (Raeven et al., 2018). Circulating small RNAs in serum EVs could be used as diagnostic markers of disease, and the reliability of the model was supported by the corresponding data (Koi et al., 2020). Systemic inflammation causes significant changes in the ratio of neutrophils and peripheral blood mononuclear cell (PBMC)-specific Y-RNA in plasma and the ratio of Y-RNA subtypes can distinguish septic plasma from healthy plasma (Driedonks and Nolte-'t Hoen, 2018; Driedonks et al., 2020).

### EVs as Drugs in Therapy

Currently, there are two mainstream insights regarding the latest treatment strategies of EVs in sepsis. a) The application of EVs derived from leukocytes, macrophages, neutrophils, mesenchymal stem cells (MSCs), and endothelial cells in the treatment of sepsis. b) Immune cells derived EVs as drug delivery carriers to treat Sepsis. EVs generated by MSC play an important role in the treatment of Sepsis and the key concept of MSC-EVs therapy is to inhibit the replication of pathogens, activate the phagocytic function and anti-inflammatory activity of macrophages, regulate immunity, repair injured tissues, and regulate the levels of anti-inflammatory and anti-inflammatory factors *in vivo*, which play an important role in the treatment of Sepsis (You et al., 2021). The paracrine effect of MSCs can realize the transfer of nucleic acids and proteins between damaged cells, and has been shown to have a beneficial effect on sepsis, thereby reducing sepsis-related morbidity and mortality (Mei et al., 2010; Weil et al., 2011; Alcayaga-Miranda et al., 2017). MSC-derived EVs can transfer mitochondria, proteins, mRNA and miRNAs *via* binding to the CD44 receptor on macrophages, which can inhibit cytokine storms but promote the production of anti-inflammatory cytokines, ATP and oxidative phosphorylation, thereby contributing to the recovery of lung injury (Karn et al., 2021). EVs released by human umbilical cord mesenchymal stem cells (huc-MSCs) inhibit the expression of inflammatory cytokine by transferring miR-377-3p (Wei et al., 2020). EVs derived from bone marrow stromal cells (BMSCs) accomplish antibacterial effects through enhancing the phagocytosis of bacterial monocytes to some extent (Alcayaga-Miranda et al., 2017). MSC-EVs have been proven to have therapeutic benefits for ARDS and severe pneumonia in reducing acute inflammation, promoting alveolar epithelial regeneration and lung endothelial cell repair (Shah et al., 2019; Su et al., 2021). Repairing injured alveolar epithelial cells is an effective therapeutic strategy to alleviate sepsis-induced ARDS (Bao et al., 2015). MSC-EVs can transfer keratinocyte growth factor (KGF) mRNA to alveolar epithelial cells, thereby restore lung proteins permeability and reduce alveolar inflammation (Zhu et al., 2014). Clinical trials have shown that MSCs and MSC-EVs can inhibit the secretion of pro-inflammatory cytokines and the accumulation of immune cells in the lungs of patients infected with COVID-19 (Su et al., 2021).

Although there are several undergoing clinical trials to examine the impact of MSC-EVs on ARDS induced by COVID-19, the application of MSC-EVs still needs further evaluation due to the limitation of clinical trial samples (Borger et al., 2020). In addition to MSCs, EVs produced by immune cells (such as DCs and macrophages, PNMs) also play an important role in the occurrence and development of sepsis and ARDS (Yanez-Mo et al., 2015). EVs derived from induced pluripotent stem cells (IPSCs) could be served as delivery vehicles for siRNAs. The application of EVs encapsulating siRNA targeting intercellular cell adhesion molecule-1 (ICAM-1) for treatment can inhibit the expression of ICAM-1 induced by LPS in recipient cells. In turn, they exert their protective effect in ALI and can inhibit the deterioration of the disease in the direction of ARDS (Ju et al., 2017). Similarly, currently engineered EVs from BMDM as a drug delivery platform can deliver siCCR2 to the spleen by intravenous administration, thereby effectively inhibiting the infiltration of some inflammatory monocytes/macrophages in the spleen, thereby alleviating the subsequent Sepsis symptoms, and such EVs have the characteristics of good biocompatibility and easy preparation (Ding et al., 2022). Incubated with paclitaxel, MSCs could release drugs in EVs-mediated way, thereby inhibiting the occurrence and development of tumors (Pascucci et al., 2014). The siRNAs in EVs can target the human epidermal growth factor receptor 2(HER2)mRNA in HEK-293T, exerting the inhibitory effect of siRNAs silencing, and inhibiting the expression of HER2, which plays an important role in the treatment of HER2^+^ breast cancer (Lamichhane et al., 2016). There is no officially recommendation for drug treatments for sepsis and sepsis-induced ARDS patients, partially due to the drugs insufficient effect on targeting the damaged organs (Gao et al., 2017). The efficiency of EVs being absorbed by their receptor cells is about 30 times that of synthetic nanoparticles, which provides advantages for a series of gradient drug dose treatments, and makes up for the lack of sufficient drug-mediated receptor cells to play Insufficient therapeutic effect (Kim et al., 2016). Therefore, EVs become potential cell-free therapy candidate against various diseases (Karn et al., 2021).

## Conclusions

Sepsis is a syndrome of physiological, pathological and biochemical abnormalities caused by infection and ARDS is a pulmonary complication involving inflammation and dysfunction of the endothelial layer. Accumulating evidences have demonstrated that EVs play critical roles in the progression of sepsis and ARDS. Different kinds of proteins and non-coding RNAs can exert pro-inflammatory or anti-inflammatory effects, which can aggravate or alleviate the disease condition. Inhibiting the infection of pathogens in the early stage can effectively inhibit the occurrence and development of the disease. EVs are an important way for pathogens to infect host cells. They can mediate the interaction between pathogens and host cells in two ways. In summary, EVs can serve as potential therapeutic targets and diagnostic indicators for sepsis and ARDS. As drug delivery carriers, EVs also have great research prospects in the treatment of sepsis and ARDS. In addition, EVs also provides new ideas for the development of new drugs related to sepsis and ARDS.

## Outlook

Currently, studies about EVs in sepsis and ARDS are mainly focused on the proteins and miRNAs. However, there are relatively few studies on the role of lipids and other metabolites, and some kinds of non-coding RNAs (such as tRNA-derived small RNAs) in sepsis and ARDS, which would attract more attention in the future. The specificity for inhibition of exact category of EVs secretion would be another barrier to demonstrate their individual downstream biological function. Moreover, methodology could be another barrier for EVs research in infectious diseases. New technologies are conducive and required to the exploration of the role of EVs in diseases (Raeven et al., 2018). The way to set up accurate diagnostic standard and whether the diagnostic markers applied to different diseases remain unclear and need further exploration and evaluation. Current studies on EV secretion kinetics during different stages of sepsis are still poorly investigated. The EVs characterization with the development stage with a specific type of EVs would be further investigated (Burgelman et al., 2021). Due to the heterogenous of components, sources and recipient cells of EVs, the clinical applications for the therapeutic dose and route of administration of EVs require extensive exploration (Abreu et al., 2021). Although EVs are used for treatment with low immunogenicity, EVs produced by different organisms may exhibit potential immune rejection factors during treatment. The safety and reliability of their efficacy is still another concern when applied to further clinical trials (Behrens et al., 2020).

## Author Contributions

WJ, HW, LZ, and WY wrote and reviewed the manuscript. WJ prepared the figure. WJ and HW summarized the tables. All authors contributed to the article and approved the submitted version.

## Funding

This work was supported by grant from the National Key Research and Development Program of China (2021YFC2501800) and Hubei Province Government Guided funding for supporting Critical Care Research (2020ZYYD004).

## Conflict of Interest

The authors declare that the research was conducted in the absence of any commercial or financial relationships that could be construed as a potential conflict of interest.

## Publisher’s Note

All claims expressed in this article are solely those of the authors and do not necessarily represent those of their affiliated organizations, or those of the publisher, the editors and the reviewers. Any product that may be evaluated in this article, or claim that may be made by its manufacturer, is not guaranteed or endorsed by the publisher.

## Glossary

**Table:**

A2MG	alpha-2-macroglobulin
ALI	acute lung injury
ARDS	acute respiratory distress syndrome
BALF	bronchoalveolar lavage fluid
BMDM	bone marrow-derived macrophages
BMSCs	bone marrow stromal cells
BPD	bronchopulmonary dysplasia
CeRNA	competing endogenous RNA
COPD	chronic obstructive pulmonary disease
CPE	choroid plexus epithelium
CRP	c-reactive protein
CRPC	castration-resistant prostate cancer
CSF	cerebro-spinal fluid
DAD	diffuse alveolar damage
DIC	disseminated intravascular coagulation
ECM	extracellular matrix
EPCs	endothelial progenitor cells
ERK	extracellular signal-regulated kinase
ESCRT	endosomal sorting complexes required for transport
ESEs	early sorting endosomes
EVs	extracellular vesicles
HER2	human epidermal growth factor receptor 2
HGF	hepatocyte growth factor
HIF	hypoxia-inducible factor
HMGB1	high mobility group box 1
HSP	heat-shock protein
HucMSCs	human umbilical cord mesenchymal stem cells
IAV	influenza A virus
ICAM-1	Intercellular cell adhesion molecule-1
ICIs	immune checkpoint inhibitors
IDCs	Immature dendritic cells
IFN-ϒ	Interferon ϒ
IL	interleukin
ILVs	intraluminal vesicles
IPMCs	intracellular plasma membrane–connected compartments
IPSCs	induced pluripotent stem cells
IRE1	inositol required enzyme 1
KGF	keratinocyte growth factor
LPS	lipopolysaccharide
LS	Lung surfactant
LSEs	late sorting endosomes
MA	Manumycin-A
MDSCs	myeloid-derived suppressor cells
MFG-E8	milk fat globule epidermal growth factor 8 factor VIII
miRNA	microRNA
MLCK	myosin light-chain kinase
MPMVECs	mouse pulmonary microvascular endothelial cells
MSCs	mesenchymal stem or stromal cells
mUC	metastatic urothelial carcinoma
MVBs	multivesicular bodies
NDMVs	neutrophil-derived micro-vesicles
NDTRs	neutrophil-derived trails
NE	neutrophil elastase
NLRP3	nucleotide-binding oligomerization like receptor 3
PBMC	peripheral blood mononuclear cell
PCT	procalcitonin
PDCD4	programmed cell death 4
PERK	PKR-like ER kinase
PIK3R2	phosphoinositide-3-kinase regulatory subunit 2
PMN	neutrophil
PS	phosphatidylserine
Rab	Ras superfamily proteins with GTPase activity
RAGE	receptor for advanced glycation end products
RPTOR	regulatory-associated protein of mTOR
SIRT1	sirtuin 1
SNARE	SNAP (soluble NSF attachment protein) Receptor
SOCS1	suppressor of cytokine signaling 1
sPLA2-IIA	Secretory phospholipase-IIA A2
STAT3	Signal transducer and activator of transcription factor
sTREM-1	soluble triggering receptor expressed on myeloid cells-1
suPAR	soluble urokinase plasminogen activator receptor
TF	tissue factor
TLR	Toll-like receptor
TNF-α	tumor necrosis factor-α
TsRNAs	tRNA-derived small RNAs
TSE	tobacco smoke extract
VCAM1	Vascular cell adhesion molecular1
VEGF-α	Vascular endothelial growth factor-α
